# The additive nature of the human multisensory evoked pupil response

**DOI:** 10.1038/s41598-020-80286-1

**Published:** 2021-01-12

**Authors:** Nathan Van der Stoep, M. J. Van der Smagt, C. Notaro, Z. Spock, M. Naber

**Affiliations:** Department of Experimental Psychology, Helmholtz Institute, Utrecht University, Langeveld Building, Room H0.26, Heidelberglaan 1, 3584 CS Utrecht, The Netherlands

**Keywords:** Psychology, Human behaviour

## Abstract

Pupillometry has received increased interest for its usefulness in measuring various sensory processes as an alternative to behavioural assessments. This is also apparent for multisensory investigations. Studies of the multisensory pupil response, however, have produced conflicting results. Some studies observed super-additive multisensory pupil responses, indicative of multisensory integration (MSI). Others observed additive multisensory pupil responses even though reaction time (RT) measures were indicative of MSI. Therefore, in the present study, we investigated the nature of the multisensory pupil response by combining methodological approaches of previous studies while using supra-threshold stimuli only. In two experiments we presented auditory and visual stimuli to observers that evoked a(n) (onset) response (be it constriction or dilation) in a simple detection task and a change detection task. In both experiments, the RT data indicated MSI as shown by race model inequality violation. Still, the multisensory pupil response in both experiments could best be explained by linear summation of the unisensory pupil responses. We conclude that the multisensory pupil response for supra-threshold stimuli is additive in nature and cannot be used as a measure of MSI, as only a departure from additivity can unequivocally demonstrate an interaction between the senses.

## Introduction

Spatial orienting is an important function of the human brain that allows us to efficiently perceive and act upon the world around us. For example, using our senses, we can quickly determine the location of an approaching car when crossing the street and adjust our actions accordingly. Our senses provide both unique and redundant information about the environment. For example, whereas both vision and hearing provide spatial information about events in the region of space in front of the body, hearing provides information about events outside of the field of view^1–3^ and vision generally allows for more accurate and precise localization of information than audition^4,5^. It is well established that the human brain can integrate information obtained via different senses, resulting in faster, more accurate, and precise orienting behaviour^5–12^. For example, audiovisual events can be detected more quickly and attracts gaze more rapidly than a purely visual or auditory event due to multisensory integration (MSI, e.g.^5^).

Various studies have demonstrated that, among others, the superior colliculus (SC, a subcortical structure) is important for integrating sensory input and generating eye-movements to unisensory and multisensory events^13–16^. The SC contains multisensory neurons that respond to input from different sensory modalities and contribute to the multisensory enhancement of, among others, orienting behaviour. The underlying neural computation of these multisensory neural responses has been characterized as linear (additive: equal to the sum of the unisensory responses), or non-linear (i.e. sub-additive: less than the sum, or super-additive: larger than the sum;^17^). Additionally, the SC is involved in transient changes in pupil size^18^. It has been suggested that the pupil’s response to sensory events plays an important role in orienting responses as it is modulated by saliency, focused spatial attention, and motor coordination^19–21^.

Given the role of the SC in MSI, spatial attention, and pupil responses, it may come as no surprise that researchers have started investigating the nature of the multisensory pupil response. A central question in many multisensory studies, which is no different in the case of the multisensory pupil response, is whether the observed multisensory behaviour is different from response to unisensory stimuli (e.g. sound or light alone). In addition, a comparison between the multisensory and the sum of unisensory responses often provides insights into the particular computation driving the multisensory behaviour as deviations from the sum of the unisensory responses can be used as a strict criterion for multisensory integration^22^.

Whether the multisensory pupil response is linear or non-linear is not trivial, as the interpretation regarding the occurrence of MSI when measuring behaviour typically depends on this outcome. In some cases, knowing whether MSI caused a certain behavioural outcome is not only relevant from a fundamental perspective, it could also be relevant for clinical applications. For example, it has been argued that being able to measure MSI using pupil response measures is especially advantageous in patient populations in which, for example, classic response time measures of MSI cannot be used (e.g.^23^). If the multisensory (pupil) response is larger (or smaller) than the linear sum of the unisensory (pupil) responses, then one can generally draw the conclusion that the multisensory response is driven by integrated sensory input and that patients can integrate sensory input. However, when the multisensory pupil response is additive, there is no behavioural evidence that indicates that MSI is the driving factor behind the multisensory pupil response. In most cases, the most parsimonious explanation would then be that the observed multisensory behaviour is the result of the independent processing of sensory input (see^24^).

So far, however, conflicting results have been reported with regard to the nature of the multisensory pupil response, which casts some doubt on whether and how the multisensory pupil response is driven or modulated by MSI. For example, previous research in monkeys has shown that the multisensory pupil response to audiovisual events is similar to the sum of the unisensory pupil responses (^25^; also see Fig. 3B showing a mixture of sub- and super-additive multisensory responses and Fig. S6B indicating (sub-)additivity in^26^). However, a more recent study in humans suggests the multisensory pupil response to be super-additive^23^. Thus, evidence regarding the nature of multisensory pupil response points in different directions. Whereas there is support for the notion that the multisensory pupil response is larger than the sum of the unisensory pupil responses^23^, other studies have shown that the multisensory pupil response is additive or sub-additive^25,26^.

Given these conflicting findings in previous studies, we investigated the nature of the human multisensory pupil response in more detail using (1) two different behavioural paradigms and (2) multiple types of visual stimuli that evoke opposite pupillary responses, and (3) only supra-threshold stimuli that evoke robust behavioural and physiological responses. In previous studies, stimulus events were either characterized by sudden onsets (^25^; our Experiment 1) or a visual stimulus changing in form (^23^; our Experiment 2). We therefore tested for MSI at a behavioural level by measuring both RTs and pupil responses in a simple detection paradigm (Experiment 1) and a visual change-detection paradigm (Experiment 2). We checked for MSI in RTs by testing for race model inequality violations^10,27–29^ and for super/sub-additivity by comparing the multisensory pupil response to the sum of the unisensory pupil responses. Second, the multisensory stimuli used in previous studies consisted of the combination of visual stimuli that either evoked pupil constriction or dilation (cf.^23,30^). Therefore, in Experiment 1, all participants were presented both with visual stimuli that either evoked a pupil dilation or constriction, auditory stimuli that evoked a pupil dilation, and the combination of these stimuli. If the multisensory pupil response is super-additive or sub-additive, then one would expect that the multisensory pupil response is larger or smaller than the sum of the unisensory pupil responses, independent of the direction of the pupil response and type of visual event. However, if the multisensory pupil response is additive, then we cannot conclude whether the multisensory pupil response to supra-threshold stimuli is driven by MSI as it can also be explained by independent processing of sensory input. The impact of the type of task was investigated by using a change detection task similar to^23^ in Experiment 2.

## Results

### Experiment 1

**Figure 1:**
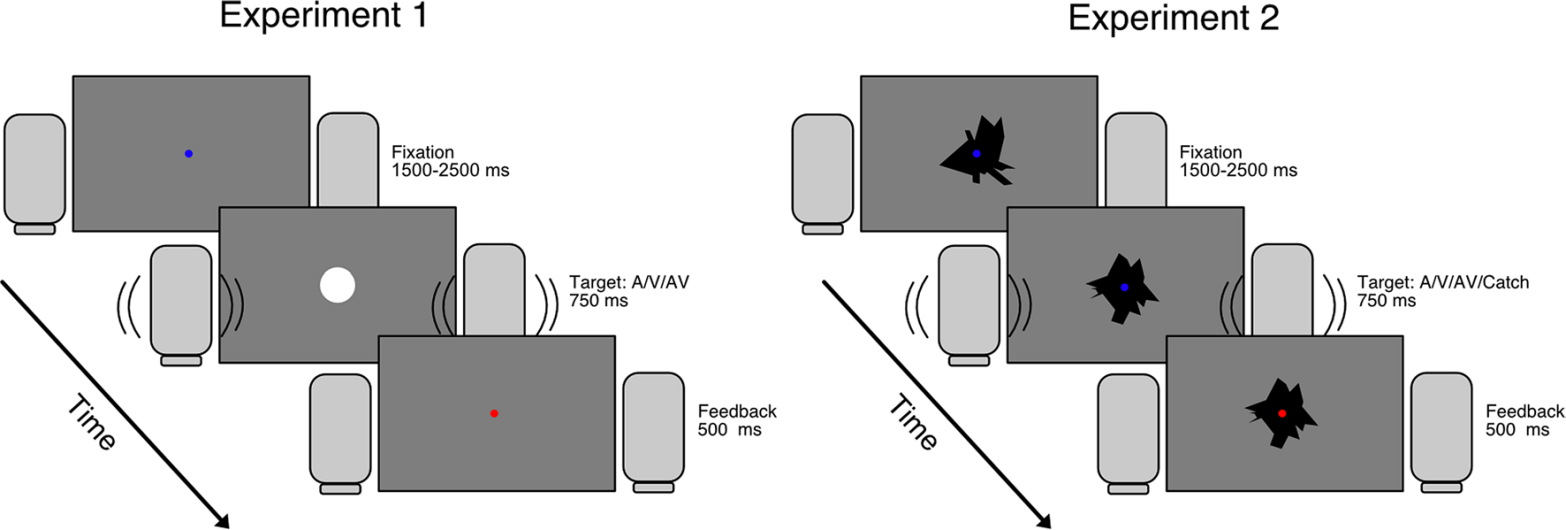
Schematic depictions of an audiovisual trial in Experiment 1 and 2.

Twelve participants were tested In Experiment 1 (see Fig. 1, left panel). Participants took part in a response and a no response block. They were instructed to respond as fast as possible to the onset of a sound or light in the response block and to only passively observe the stimuli in the no response block. In both types of blocks pupil responses were recorded.

### Hits

The proportion of hits was very high in all conditions (range across all conditions = 0.92–1). Therefore, we did not analyse the proportion of hits further.

### Response time data

Response times were only collected in the response block.

### Response times

**Figure 2:**
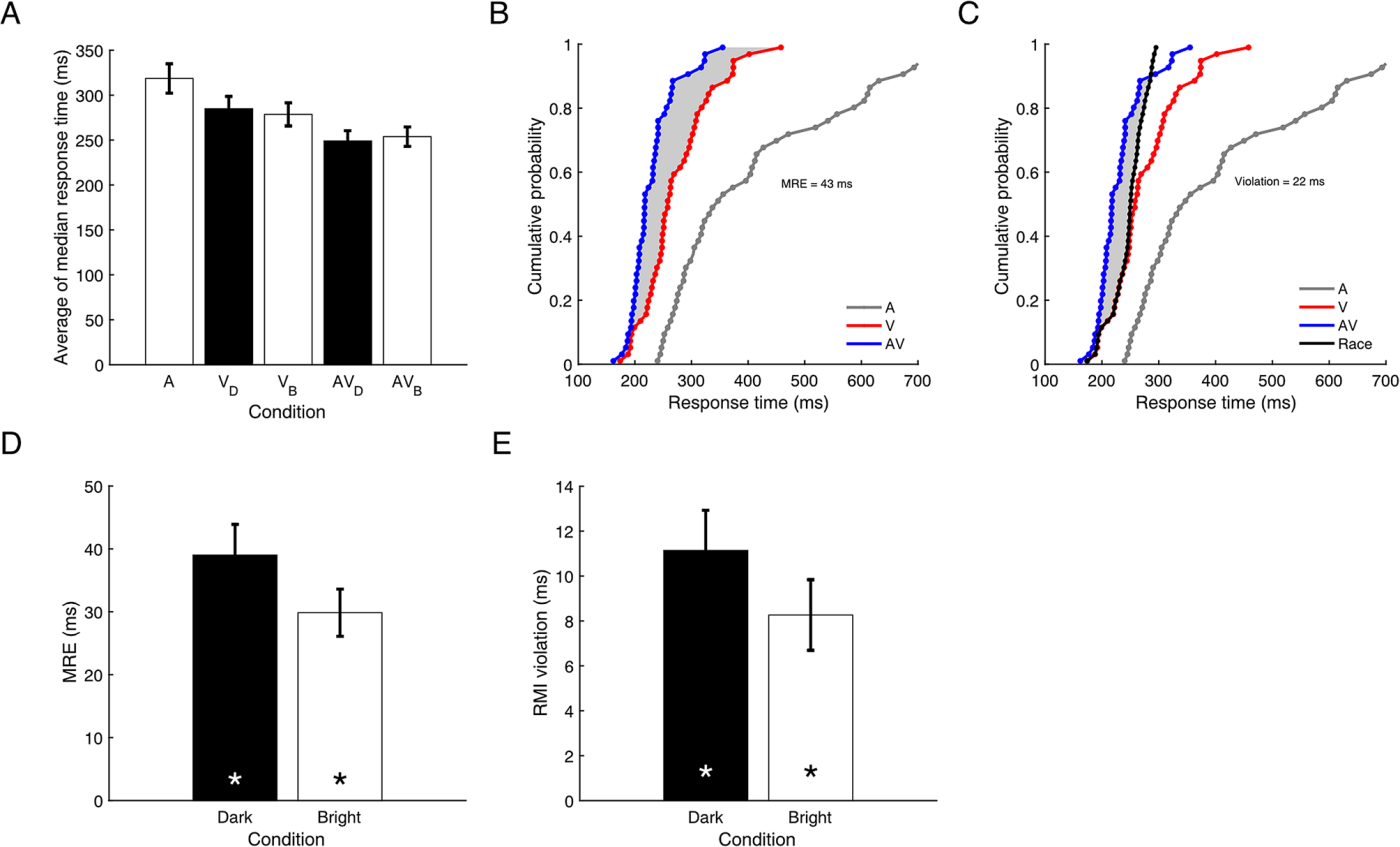
(**A**) The average of the median response times in the auditory (A), visual dark (V_D_), visual bright (V_B_), audiovisual dark (AV_D_), and audiovisual bright (AV_B_) condition in the Response block in Experiment 1. Significant differences between conditions are not indicated in panel (**A**). See the text for information on RT differences. (**B**) A single-participant example of the area between Grice’s bound^31^ and the AV CDF (in grey) indicating the amount of MRE. (**C**) A single-participant example of the area (grey shading) between the race model (Miller’s bound^10^, black line) and the AV CDF (blue line) indicating the amount of RMI violation. (**D**) The amount of multisensory response enhancement for bright and dark AV targets. (**E**) Race model inequality violation for bright and dark targets. Error bars indicate standard errors. Asterisks indicate BF_+0_ > 200.

It was first determined whether participants responded faster to multisensory stimuli than unisensory stimuli. This was indeed the case, both for bright and dark stimuli (Fig. 2A). A Bayesian repeated measures Analysis of Variance (ANOVA) for RTs in the dark target condition indicated very strong evidence for an effect of Target Modality compared to a null model assuming no effect (A, V_Dark_, AV_Dark_; BF_10_ = 625,233; Note that BF_10_ indicates evidence in favour of the alternative hypothesis and BF_01_ evidence in favour of the null-hypothesis). Post-hoc tests corrected for multiple testing indicated that responses in the AV_Dark_ condition (*M* = 249 ms, *SD* = 40) were faster than in the V_Dark_ (*M* = 285 ms, *SD* = 47, BF_10_ = 2356) and A condition (*M* = 319 ms, SD = 57, BF_10_ = 9908). Responses in the V_Dark_ condition were also faster than responses in the A condition (BF_10_ = 22.545).

Another Bayesian repeated measures ANOVA was conducted using the bright target conditions (A, V_Bright_ and AV_Bright_). Again, there was very strong evidence for a main effect of Target Modality (BF_10_ = 153,882). Responses in the AV_Bright_ (*M* = 254, *SD* = 37) condition were faster than in the V_Bright_ condition (*M* = 279, *SD* = 45, BF_10_ = 1978) and the A condition (*M* = 319 ms, *SD* = 57, BF_10_ = 1625). RTs in the V_Bright_ condition were significantly shorter than in the A condition (BF_10_ = 62.468).

### Multisensory response enhancement

To determine the amount of speed-up in the multisensory condition relative to the fastest unisensory condition, the amount of multisensory response enhancement (MRE) was analysed (grey area Fig. 2B, see “Methods” section for more information). In line with the RT analysis, Bayesian one-sample t-tests provided very strong evidence of MRE being larger than zero in the bright (*M* = 30 ms, *SD* = 13, BF_10_ = 3164, δ = 2.08) and dark AV target condition (*M* = 39 ms, *SD* = 17, BF_10_ = 3285, median δ = 2.09). There was only anecdotal evidence for a difference in the amount of MRE between the dark and bright condition (BF_10_ = 2.469, δ = 0.602, see Fig. 2D).

### Race model inequality violation

Responses to multisensory stimuli can become faster than responses to unisensory stimuli due to statistical facilitation (i.e. independent processing of sensory input). That is, the probability of fast responses simply increases when a participant can respond to sound or light when they are presented together and the observed unisensory response time distributions overlap. To investigate whether the observed MRE could be explained by statistical facilitation, the cumulative response time distribution in the multisensory condition (the blue line in Fig. 2C) was compared to the sum of the unisensory cumulative RT distributions (the race model, the black line in Fig. 2C, see “Methods” section for more information). If responses in the multisensory condition are faster than the upper limit of the race model, the race model inequality (RMI, see “Methods” section) is violated. This means that multisensory response enhancement cannot be explained by independent processing of sensory input, which is indicative of MSI (though see^11,12,29^ for considerations). Bayesian one-sided one-sample t-tests indicated there was strong evidence for the amount of RMI violation (i.e. the grey violation area in Fig. 2C) being larger than zero in both the Dark and Bright condition (see Fig. 2E). RMI violation was observed both for dark (*M* = 11.25 ms, *SD* = 6.384, BF_+0_ = 725, median δ = 1.565) and bright AV targets (*M* = 8.25 ms, *SD* = 5.512, BF_+0_ = 223, median δ = 1.316). There was no evidence for or against a difference in violation area between the Dark and Bright condition (BF_10_ = 0.651, median δ = 0.337).

Overall, these results are indicative of MSI in the Bright and Dark target condition as the observed multisensory response enhancement cannot simply be explained by independent processing of sensory input.

## Pupillometry data

**Figure 3:**
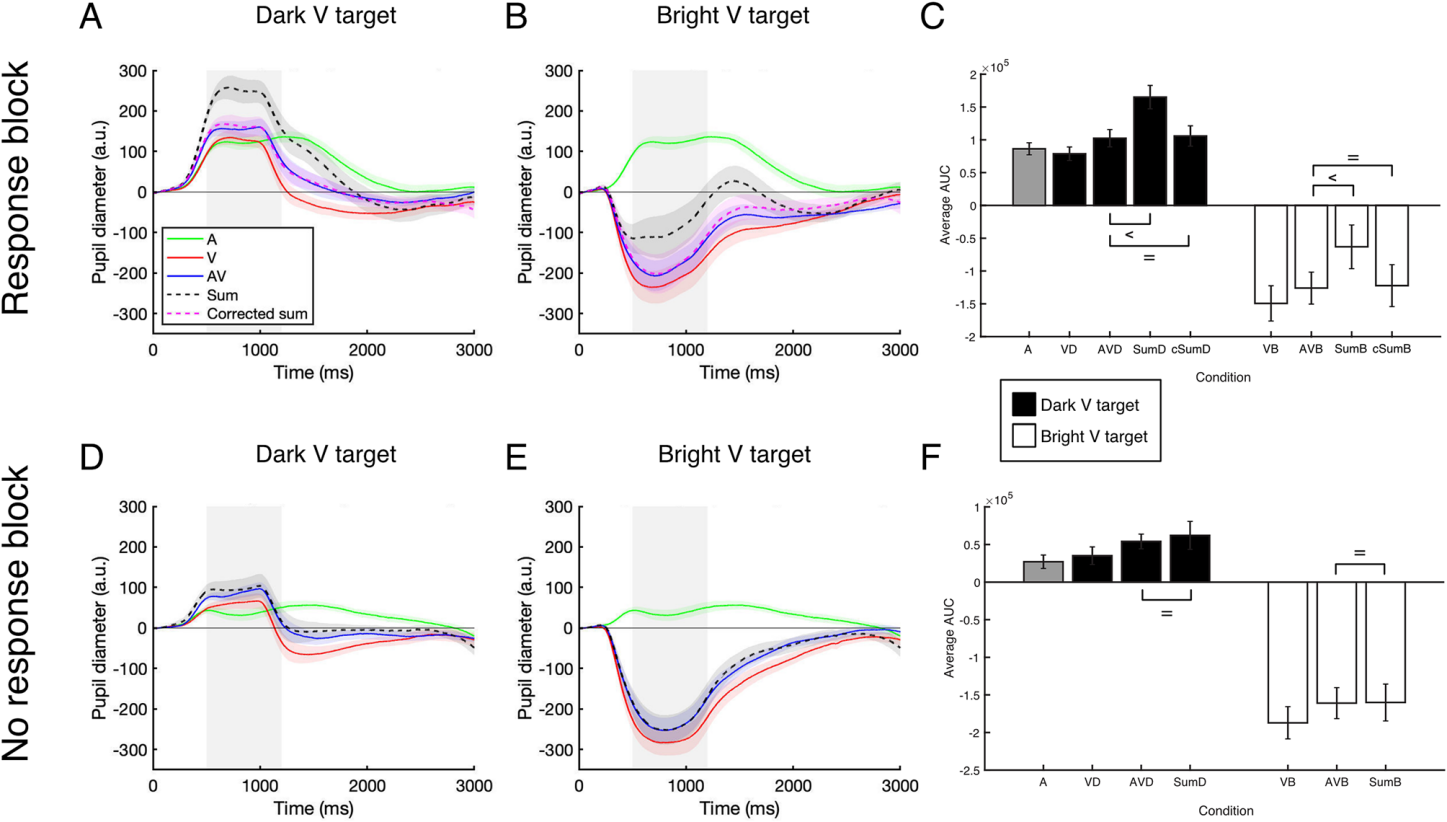
The average pupil traces and average area under the curve for the selected time window (grey area) for auditory (green), visual (red), audiovisual, (blue), and the (corrected) sum of auditory and visual pupil responses (purple and black dashed line) in the response (panel **A**–**C**) and no response block (panel **D**–**F**) for dark and bright targets. Corrected means that one motor-evoked pupil response is subtracted from the sum of unisensory pupil responses. *cSumD *corrected sum dark, *cSumB *corrected sum bright. Shaded areas around the pupil traces in panels (**A**, **B**, **D**, **E**) and error bars in panels (**C**, **F**) indicate standard error of the mean. The “equals” sign indicates BF_01_ > 2.9, the “less than” sign indicates BF_10_ > 80.

To investigate the nature of the multisensory pupil response, the pupil response to AV targets was compared to the sum, and in the Response block also to the *corrected sum*, of the unisensory pupil responses (see Fig. 3). The *corrected sum* is the sum of the pupil responses to auditory (A) and visual (V) stimuli, from which the pupil response related to a button press was subtracted. This in order to more fairly compare the AV and summed pupil responses. The amount of subtraction (i.e. correction) was calculated by subtracting the pupil response in the no response from the response block for auditory stimuli (see “Methods” section and Supplementary Fig. 1).

Bayesian paired samples t-tests were conducted to test for differences in the area under the curve (AUC) of the pupil response from 500 to 1200 ms after stimulus onset between the AV condition, the sum (A + V), and the corrected sum (see Fig. 3).

In the Response block, the pupil response to AV stimuli showed subadditivity when compared to the sum of A and V both for dark targets (BF_10_ = 7688, median δ = − 2.317) and bright targets (BF_10_ = 88, median δ = − 1.268). This could be interpreted as a deviation from additivity (i.e. sub- or super-additivity, see Fig. 3A,B). However, when the pupil response to AV targets was compared to the corrected sum (A + V – (A_Response_ – A_No response_)), there was no difference between the AV and the corrected sum of the unisensory pupil responses in both the dark (BF_01_ = 3.092, median δ = − 0.122) and bright condition (BF_01_ = 3.377, median δ = − 0.061, compare the purple dashed line and the blue line in Fig. 3A,B, and see the AV versus cSum comparisons in Fig. 3C). This demonstrates additivity of the AV pupil response for both dark and bright AV targets in the response block. Similarly, the results from the no response block indicate that the pupil response to AV targets was more likely to be similar to the (in this case uncorrected) sum of the unisensory pupil responses for both dark (BF_01_ = 2.971, median δ = − 0.141) and bright targets than different from the sum (BF_01_ = 3.463, median δ = − 0.024, compare the black dashed line and the blue line in Fig. 3D,E, and see the AV versus Sum comparisons in Fig. 3F; see Supplementary Figures S1–S3, for the same figures but corrected for the motor component in the visual rather than auditory pupil response; see Supplementary Figure S4 and S5 for the sequential analyses).

## Discussion Experiment 1

In this first Experiment, the response time analysis in the response block suggests that MSI had occurred with these stimuli as shown by significant RMI violation. We also observed that the multisensory pupil response was different from the unisensory pupil responses but always equal to the (corrected) sum of the unisensory pupil responses. This indicates that the multisensory pupil response is additive in nature, both when targets had to be responded to and when passively viewed. This is true both for pupil dilation and constriction responses. This means that although the response time behaviour seems to be driven by MSI, the multisensory pupil response can be explained by summation of the independent effects of unisensory signals. Based upon these results one could argue that, given that the multisensory pupil response cannot be distinguished from the sum of the unisensory responses, the multisensory pupil response may not be a good candidate for measuring MSI in populations for which RT data collection is not feasible. However, although the simple detection task used in Experiment 1 is a classic paradigm for measuring MSI, it is different from the paradigm used in a recent study that did show super-additivity of the multisensory pupil response^23^. Thus, to further investigate the nature of the multisensory pupil response, we used a change-detection paradigm in Experiment 2 that was similar to that of Rigato et al.^23^. Yet, in their study, pupil responses and response times were measured in different blocks and never together in the same block, while in Experiment 2, we again used a response and a no response block and measure pupil changes and response times simultaneously in the response block.

### Experiment 2

In this second experiment, twelve participants were tested in a response and a no response block while their pupil size was recorded (see Fig. 1, right panel). In the response block, they were instructed to respond as fast as possible to a change in the shape of a visual stimulus or the onset of an auditory stimulus, and withhold their response when no visual change nor a sound was presented. In the no response block they were instructed to passively view and listen to the stimuli.

### Hits

The proportion of hits was very high in all conditions (range across all conditions = 0.875–1). The average proportion of correctly withheld responses during catch trials was 1 (*SD* = 0).

## Response time data

### Response times

**Figure 4:**
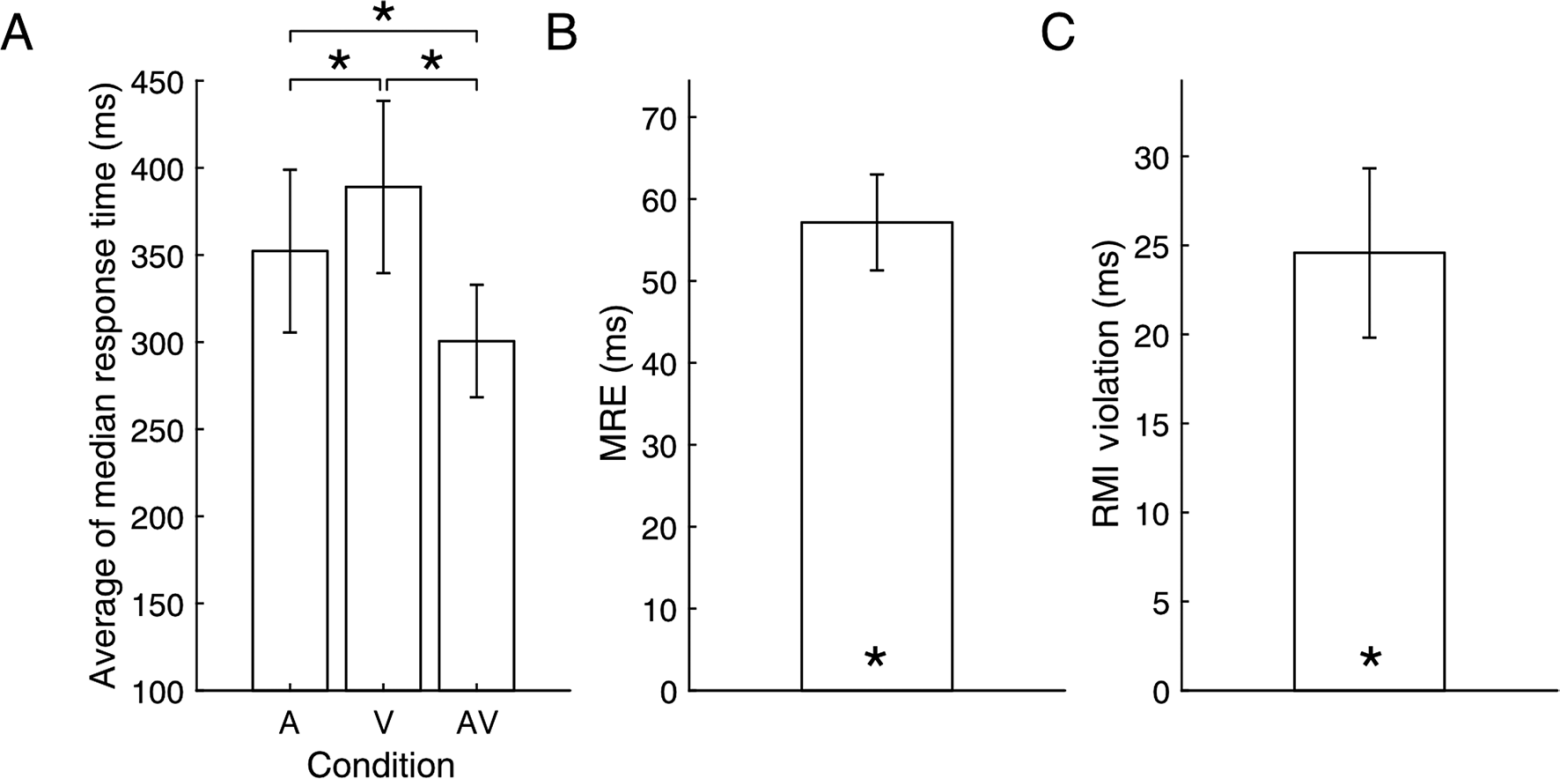
(**A**) The average of the median response times in the auditory (A), visual (V), and audiovisual (AV) condition in Experiment 2. (**B**) Multisensory response enhancement. (**C**) Race model inequality violation. (**D**) Error bars indicate standard errors. Asterisks indicates BF_10_ > 24.

The average of the median RTs in each condition is shown Fig. 4A. A Bayesian repeated measures ANOVA for RTs indicated there was very strong evidence for a main effect of Target Modality (BF_10_ = 4.107E+6) compared to a null model assuming no effect. Bayesian post-hoc tests corrected for multiple testing indicated that responses in the AV condition (*M* = 301 ms, *SD* = 32) were significantly faster than in the V (*M* = 389 ms, *SD* = 49, BF_10_ = 28,558) and A condition (*M* = 352 ms, *SD* = 47, BF_10_ = 1202). Responses in the A condition were faster than responses in the V condition (BF_10_ = 25).

## Multisensory response enhancement

There was strong evidence that the average amount of MRE was significantly different from zero (*M* = 57 ms, *SD* = 20, BF_10_ = 17,035, median δ = 2.546, see Fig. 4B).

## Race model inequality violation

To test whether the observed MRE could be explained by statistical facilitation, the amount of RMI violation was compared to zero using a one-sided Bayesian one-sample t-test. As expected, there was strong evidence of violation of the RMI (*M* violation = 25 ms, *SD* = 17, BF_+0_ = 200, median δ = 1.294), indicative of an interaction between the senses (see Fig. 4C).

## Pupillometry data

**Figure 5:**
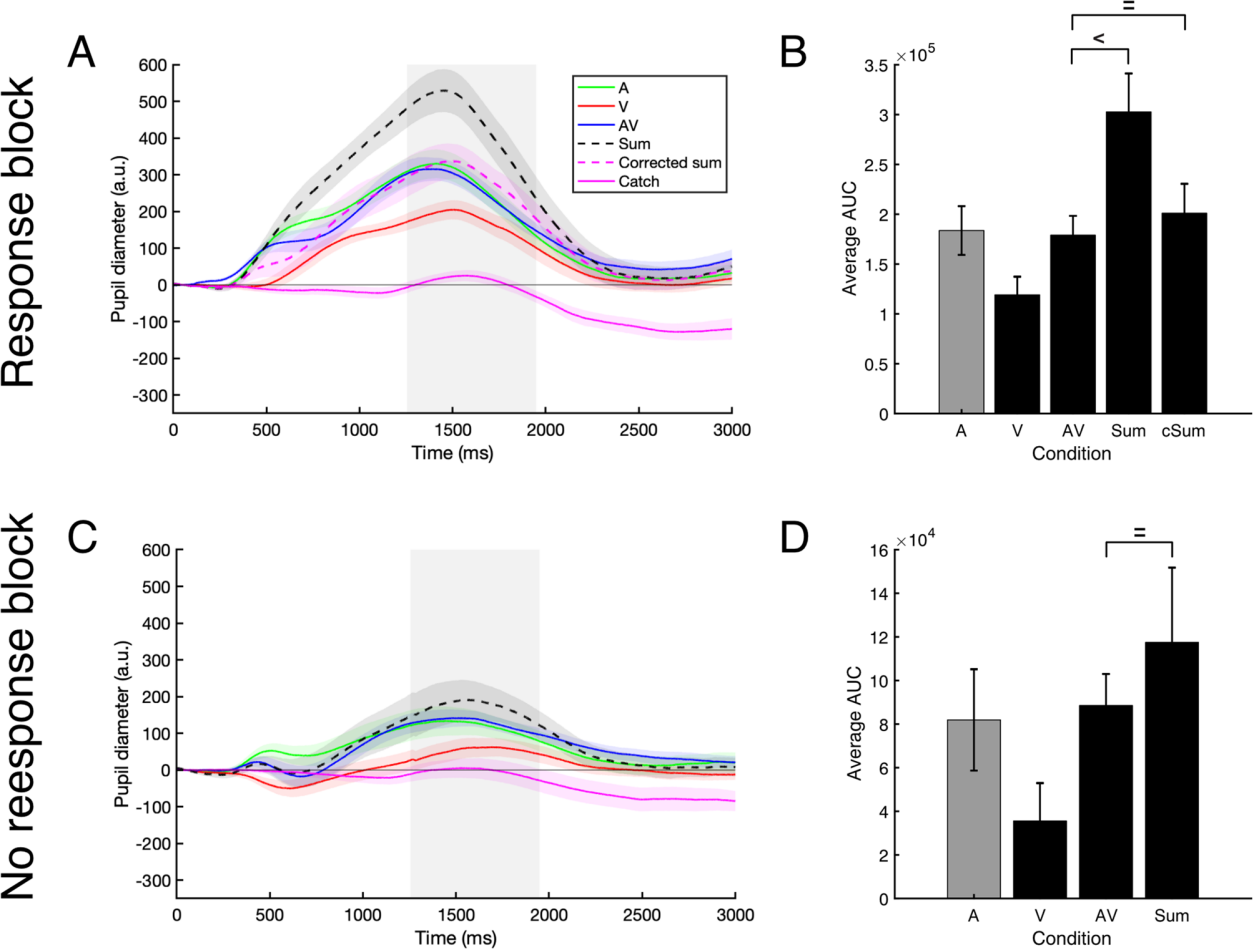
The average pupil traces and average area under the curve for the selected time window (grey area) for auditory (green), visual (red), audiovisual (blue) targets, and the (corrected) sum of auditory and visual pupil responses (purple and black dashed line) in the response (panel **A** and **B**) and no response block (panel **C** and **D**), and for catch trials (purple). Shaded areas around the pupil traces in panels **A** and **C** and error bars in panels **B** and **D** indicate standard error of the mean. The “equals” sign indicates BF_01_ > 1.6, the “less than” sign indicates BF_10_ > 14.

Bayesian paired-samples *t*-tests on the AUC between 1255 and 1948 ms of the pupil trace post stimulus onset (the significant time window in^23^ indicated that that there was strong evidence that the audiovisual pupil response in the response block was smaller than the sum of the unisensory pupil responses (BF_10_ = 15, median δ = − 0.927, compare the black dashed line and the blue line in Fig. 5A). The pupil response in the AV condition was more likely to be similar to the corrected sum of the unisensory responses than different (BF_01_ = 2.836, median δ = − 0.161, compare the purple dashed line and the blue line in Fig. 5A and see Fig. 5B). In the no response block, the audiovisual pupil response was more likely to be similar to, rather than different from, the sum of the unisensory responses (Bayesian Wilcoxon Signed-Rank test due to violation of normality, BF_01_ = 1.64, median δ = − 0.260, compare the black dashed line and the blue line in Fig. 5C and see Fig. 5D; see Supplementary Figs. S6 and S7, for the same figures but corrected for the motor component in the visual rather than auditory pupil response; see Supplementary Fig. S8 for the sequential analyses).

Overall, the results from Experiment 2 also indicate that the multisensory pupil response was additive in nature.

## General discussion

The integration of sensory information from multiple senses is known to lead to a plethora of behavioural benefits such as faster detection and more accurate perception of stimuli^3,32,33^. In addition to changes in overt behaviour in response to multisensory stimuli, many studies have shown differences in the (neuro)physiological responses to multisensory and unisensory stimulation (e.g.^34^). Given the important role of the superior colliculus in multisensory integration, spatial orienting, and pupil dilation^13–16,18^, we set out to investigate the nature of the multisensory pupil response. In previous studies, different effects of multisensory stimulation on the pupil response have been observed. Whereas in some studies it was observed that the multisensory pupil response is equal to the sum of the unisensory pupil responses^25^, in another study super-additive multisensory pupil responses were observed^23^. There were several differences between these studies that may have contributed to the contrasting findings. Here, we not only used two paradigms that were previously used in these studies, but also visual stimuli that evoked pupil constriction or dilation to further investigate the nature of the multisensory pupil response. More specifically, we investigated whether the multisensory pupil response was similar to or different from the sum of the unisensory pupil responses. Our findings show that the multisensory pupil response is different from either of the unisensory pupil responses, but equal to the linear sum of the unisensory pupil responses in all experiments and conditions when supra-threshold stimuli are used.

Although multisensory neurons in the superior colliculus may potentially have contributed to the observed multisensory pupil response, Occam’s razor would lead us to conclude that the observed multisensory pupil responses reflect the sum of independent sensory processes. Unfortunately, the link between multisensory neuronal activity and multisensory behaviour is not straightforward, which complicates this argument. Multisensory neurons vary in their response properties, and the response of a single multisensory neuron can vary from sub-additive, to additive, and super-additive depending on the stimulus effectiveness^35^. It is unclear how the activity of all multisensory neurons in the SC together drive multisensory behaviour. If the average response of multisensory neurons in the SC is additive this might still lead to multisensory facilitation of motor responses and to an additive multisensory pupil response. However, from a behavioural point of view, we can only check whether behaviour can be described by the linear sum of unisensory pupil responses or not. If the multisensory pupil response is indeed the same as the sum of the unisensory pupil response, then this most likely reflects independent effects of the senses. Knowing whether the multisensory pupil response is driven by MSI is not trivial: For behavioural measurements, only a deviation from additivity allows a conclusive argument that multisensory integration has occurred (and thus whether the pupil response can be used as a measure of MSI). We did find support for MSI in terms of the speed of responding to the same multisensory stimuli that evoked the pupil responses as evidenced by violations of the race model inequality. This was true both for audiovisual targets containing a bright and a dark visual stimulus relative to the stimulus background. Our observation that the multisensory pupil response to supra-threshold stimuli is additive in nature is in line with previous observations in monkeys^25,26^, but in contrast with Rigato et al.^23^.

The difference between our results and Rigato et al.’s^23^, in which a super-additive multisensory pupil response was observed, could be explained by the paradigm that was used (this only applies to our Experiment 1), the intensity of the stimuli (high vs. low,the exact intensities were not reported by Rigato et al. though), and the type of sounds (white noise vs. pure tones). The most likely cause of the different results is the intensity of the stimuli used. It has been shown that the *relative* increase in a multisensory neuron’s response to multisensory input, as compared to the same neuron’s response to unisensory stimulation, is greater the weaker those unisensory responses are (see e.g., Fig. S8 in^35^). Using near-threshold stimuli may result in super-additive multisensory pupil responses. It might be important to use near-threshold stimuli for all stimuli involved (e.g. sound and light) as Wang et al.^26^ used relatively low intensity auditory stimuli and did not find super-additive multisensory pupil responses (see^26^, Fig. 3A; though additivity was not formally assessed). Using “intermediate” visual contrasts, Wang et al.^25^ also did not observe super-additivity. How stimulus contrast (and thus effectiveness, and stimulus processing time) influences the nature of the multisensory pupil response requires a more thorough investigation using multiple stimulus intensities. Additionally, given the importance of spatial and temporal proximity of audiovisual stimuli for MSI, manipulating spatial and temporal alignment (i.e., a difference in auditory and visual stimulus location and timing) could be helpful in furthering our understanding of the processes that drive the multisensory pupil response (see for example^25^).

An alternative explanation for the absence of super-additivity of the multisensory pupil response to supra-threshold stimuli in the current study could be the presence of a ceiling effect. The pupil response to a unisensory stimulus could already be close to or at the maximum dilation, not allowing a stronger dilation for multisensory stimuli. In that case, if the multisensory pupil response *is* super-additive in nature, observing a multisensory pupil response that is smaller than the sum of the unisensory responses could lead to the incorrect conclusion that the multisensory pupil response is sub-additive or additive in nature. However, we are not concerned about such a ceiling effect in our study for two reasons: (1) We observed additivity both in the response blocks (which contained larger pupil dilations than in the no response block), and in the no response blocks (which contained much smaller pupil responses). If a ceiling effect was a problem in the response block than certainly not in the no response block in Experiment 1; (2) We observed additivity both when audiovisual targets contained a visual component stimulus that evoked pupil dilation and when the visual component stimulus evoked pupil constriction. When the auditory stimulus triggers pupil dilation and the visual stimulus pupil constriction, the multisensory pupil does not have to exceed the visual or auditory evoked unisensory pupil response to be non-linear in nature. Therefore, we do not think that the observed additivity is due to a ceiling or floor effect.

The observed multisensory response enhancement and race model inequality violation in Experiment 1 and 2 is in line with previous studies of multisensory integration using response times as a dependent variable^10,36–40^. Although multisensory response enhancement can be the result of various processes (response preparation; cross-modal spatial attention; multisensory integration; switch costs, see^12,37,41–44^), the paradigm used in the current study makes is very likely that the observed RT effects are due to an interaction between the senses. Whether or not the multisensory response enhancement effects observed in the lab reflect sensory processing in more complex situations in daily life remains to be seen. For example, Corneil et al.^45^ investigated the influence of scene complexity on multisensory response enhancement of saccades and showed that the spatial properties of saccades are mainly driven by visual input and the temporal properties of the saccades by auditory input. These results may suggest that multisensory response enhancement is not always present in all circumstances. With regard to manual responses, however, it has been shown that race model inequality violation is quite robust and not affected by stimulus and environment complexity in virtual reality^46^. It could be interesting to see how the multisensory pupil response is affected by scene complexity and whether this would change the nature of the multisensory pupil response.

In sum, the current study sheds more light on the nature of the multisensory pupil response. Based on the current findings from two experiments using two different paradigms, we conclude that the multisensory pupil response is additive in nature for supra-threshold stimuli. The multisensory pupil response, although clearly different from the unisensory responses, is equal to the sum of the unisensory pupil responses in two different paradigms with response time analyses indicating an interaction between the senses. Pupil responses have been related to many phenomena ranging from changes in low-level stimulus properties such as brightness to more cognitive influences such as visual attention. Given that the multisensory pupil response can most easily be explained by linear summation of independent sensory input, while the reaction time measures are indicative of multisensory integration, it appears unwise to use pupillometry as a measure for MSI in populations (such as infants) that are unable to give a behavioural response. The intermediate layers of the Superior Colliculus are known to be involved in MSI^16,47^ and have been linked to transient changes in pupil dilation^30^, making this brainstem area a likely candidate for the origin of these effects. Regardless of whether or not the multisensory pupil response is the result of multisensory integration in the superior colliculus, the finding that the pupil seems to reflects a summation of independent processes^21^ highlights pupillometry as an ideal tool to investigate the independent contributions of different sensory processes to the pupil’s response.

## Methods

### Experiment 1

#### Participants

Twelve participants (9 female, *M*_*age*_ = 19 years, *SD* = 0.853) took part in this study. The sample size was determined based on previous studies of multisensory integration using response times (e.g.,^37^). All participants reported normal or corrected-to-normal visual acuity and normal hearing. Informed consent was obtained from each participant before the start of the experiment. The participants received money or course credits for their participation. The study protocols were approved by the faculty ethics review board (FERB) of Utrecht University (FETC18-048 Stoep) and the experiment was performed in accordance with the relevant guidelines and regulations.

## Materials and apparatus

Auditory stimuli consisted of an 80 dB(A) 750 ms white noise burst presented simultaneously from two speakers, positioned to the left and right of the monitor. This created the percept of a sound originating from the center of the screen. Visual stimuli were white (320 cd/m2) or dark grey (51 cd/m2) filled circles (2 visual degrees in diameter) presented in the centre of a grey background (132 cd/m2) for 750 ms. A small fixation dot (0.05 visual degrees in diameter) was presented at the centre of the screen throughout the experiment. The monitor was an LED Asus ROG swift monitor (AsusTek Computer Inc., Taipei, Taiwan) that displayed 2560 by 1440 pixels on a 60 cm by 35 cm flat surface. The monitor was linearized, meaning that software’s RGB input was linearly related to the screen’s luminance output (i.e., a gamma of 1 instead of 2.2). The auditory and visual stimulus were presented at the same time to create an audiovisual stimulus. An eye-link 1000 (SR Research) was used to collect eye-position and pupil size data of the right eye with a 1000 Hz sampling rate. Responses times were collected using a QWERTY keyboard connected to a PC running Matlab (Mathworks, 2019) with Psychophysics Toolbox^48,49^.

## Procedure

Participants were seated behind the monitor in a dimly lit room and had to place their chin in a chin-rest to maintain gaze at a fixed distance of ~ 55 cm. The eye-tracker was calibrated using a 9-point calibration procedure after which the participants could read the instructions, provide informed consent, and start the experiment. The experiment was divided in four blocks that alternated between two types: an active block in which participants had to respond as fast as possible to any stimulus that appeared (A, V, or AV), and a passive block in which participants had to passively watch the stimuli. Each block contained five conditions: Auditory only, bright visual stimulus, dark visual stimulus, audiovisual (bright), and audiovisual (dark). Each condition contained 50 trials, adding up to a total of 500 trials (250 in the passive and 250 in the active block). The order of blocks (passive (P) and active (A); APAP or PAPA) was counter balanced across participants. Each trial started with the presentation of a grey screen with a small, blue fixation dot for a random duration chosen from an interval between 1500 and 2500 ms. Next, the visual and/or auditory stimulus was presented for 750 ms. The trial was stopped and fixation dot turned red for 500 ms if the participant did not respond within 750 ms, otherwise the fixation dot stayed blue. See Fig. 1 for an overview of the experimental design.

## Data preprocessing

### Response time data

Response times (RTs) shorter than 120 ms were removed from further analysis because they were considered to be the result of anticipation. Given that RT distributions are generally skewed, the median response time of each condition was used rather than the average response time.

In addition to analysing median RTs, multisensory response enhancement (MRE, also called the redundant signals effect or the redundancy gain) was calculated using the RSE-box^27^ for MATLAB (2019). This measure expresses the amount of speed up in the multisensory condition relative to the fastest unisensory condition. MRE was calculated based on the cumulative distribution functions of the auditory (A), visual (V), and audiovisual (AV) conditions. First, RT outliers were removed at an individual participant and condition level using the absolute deviation around the mean as a criterion^27,50^; using the outCorrect function). RTs in each condition were then down-sampled to the number of RTs in the condition with the least amount of trials left after outlier correction (i.e. the minimum of the number of trials in the A, V, and AV condition; using the sampleDown function; see the RSE-box functions for more details). Next, the amount of MRE in the dark and bright condition was calculated using the respective unisensory and multisensory conditions. The getGain function was used to calculate the area between the AV CDF and the fastest of the two unisensory CDFs (i.e. Grice’s bound^31^, see Eq. (1) and see Fig. 2B for an example). Grice’s bound can be calculated using the following formula:

1 $$P(RT_{Grice} < t) = max \left( {P\left( {RT_{A} < t} \right), P\left( {RT_{V} < t} \right)} \right)$$

Grice’s bound is equal to maximum probability (P) of unisensory A and unisensory V response times (t), with t ranging from 0 to 1000 ms (see^1^ for more details about these procedures). The probabilities can be estimated using the observed unisensory CDFs which can then be compared to the AV CDF.

Observing MRE does not necessarily mean that auditory and visual input was integrated. Given that participants can respond to the onset of both a sound or a light, responses in the AV condition can simply be faster because the probability of a fast response increases with more stimuli to respond to. This is called statistical facilitation and its upper limit can be calculated using the right part of Eq. (2) (Miller’s bound). Violations of the race model inequality (RMI^10,28,29^) is indicative of sensory interactions:

2 $$P\left({RT}_{AV}<t\right)\le P\left({RT}_{A}<t\right)+ P\left({RT}_{V}<t\right)$$

The left part of the inequality describes the probability (P) of a given RT in the AV condition that is smaller than a certain time (t), with t ranging from 0 to 1000 ms. The right part describes the sum of the probability of a given RT in a unisensory condition that is smaller than a certain time. If auditory and visual input is independently processed, then the probability of an RT in the multisensory condition should be equal to or smaller than the sum of the probabilities of that RT in the unisensory conditions for any timepoint. If this inequality is violated (i.e. P(RT_AV_ < t) > P(RT_A_ < t) + P(RT_V_ < t)), then this indicates that some sort of interaction between the senses must have occurred. The CDFs of the observed RTs in the A, V, and AV condition were used to test the RMI. To obtain a single measure of RMI violation for the dark and bright condition per participant, the getViolation function from the RSE-box was used to calculate the violation area between the AV CDF and the race model (the sum of the A and V CDF, see Fig. 2C for an example). This was done after the same outlier correction and down-sampling steps as for the MRE measure.

## Pupillometry data

Periods of missing pupil data due to blinks were interpolated with a cubic fit. Pupil diameter, measured as an arbitrary unit, was analysed with an event-related approach, sectioning pupil time traces from stimulus onset till 3000 ms after stimulus onset. Pupil time traces were baseline corrected per trial by subtracting the average pupil size within a window of 100 ms centered around stimulus onset. Pupil diameter exhibited artefacts in some trials (e.g., due to head movements). A trial was excluded from analysis when the variance in pupil diameter over time exceeded four standard deviations from the median across all trials.

In the case of multisensory pupil responses, but also in the case of multisensory event related brain potentials^51^, one has to take into account that the sum of unisensory responses may be contaminated with factors such as arousal, expectancy, and/or motor related activity that affect all pupil responses^52,53^. This result is an ‘unfair’ comparison between responses to audiovisual events and the sum of responses to unisensory events given that the sum contains twice the arousal/expectancy/motor related activity. To account for this, a certain proportion of catch trials is often included in which no target is presented. The pupil response in this catch condition reflects the arousal/expectancy/motor related component, which can then be used to correct the linear sum of unisensory responses, allowing a better comparison of unisensory and multisensory responses (see^26^). In Experiment 1 and 2, we accounted for ‘contaminating’ effects (e.g. arousal/expectancy/motor related activity) using the pupil response to unisensory stimuli in the active (response) and passive (no-response) block in both experiments. By subtracting the unisensory (visual or auditory) pupil response in the passive block from the unisensory response in the active block we obtained an estimate of the contaminating effects (see Supplementary Figure S1 and S6). This allowed for correction of the sum of unisensory pupil responses in the active (response) block and investigate multisensory integration effects when simultaneously measuring RTs and the pupil response. We used the correction based on the difference between the pupil responses to auditory targets in the response and no response block.

To allow comparison of the pupil size between conditions, we calculate the area under the curve of the pupil response in a time window that was centred around the maximum pupil change (500–1200 ms after stimulus onset).

## Statistical analyses

The data of Experiment 1 and 2 were analysed using Bayesian methods in JASP (JASP Team, 2020). Response time data was analysed using a Bayesian repeated-measures Analysis of Variance (ANOVA) with the factor Target Modality (A, V, AV) for both bright and dark target conditions separately. The main effect was compared to a null model. Bayesian post-hoc tests were used to compare the levels within a factor.

Multisensory response enhancement was compared to zero using a two-sided Bayesian one-sample t-test and between the bright and dark condition using a Bayesian paired samples *t*-test. We used a two-sided test because both positive and negative MRE can occur and reflect facilitation and inhibition, respectively. Race model inequality violation was compared to zero using a one-side Bayesian one-sample t-test (one-sided given the nature of the inequality) and compared between the bright and dark condition using a Bayesian paired samples t-test.

Pupillometry data was compared between conditions (AV vs. sum and corrected sum) using Bayesian paired samples t-tests. A Cauchy prior with a default width of 0.707 was used in all Bayesian one-sample and paired sample *t*-tests.

### Experiment 2

All methodological aspects of Experiment 2 were identical to Experiment 1, except for the participants, stimuli, and task. A new group of twelve participants (9 females, *M*_*age*_ = 21 years, *SD* = 3.085) took part in this study. All participants reported normal or corrected-to-normal visual acuity and normal hearing. Informed consent was obtained from each participant before the start of the experiment. The participants received money or course credits for their participation. The study protocols were approved by the faculty ethics review board (FERB) of Utrecht University (FETC18-048 Stoep) and the experiment was performed in accordance with the relevant guidelines and regulations.

Per trial a stimulus was chosen from a set of 9 different stimuli consisting of black filled polygons with 25 intersections (26 edges) (see Supplementary Fig. S9). The locations of its intersections were determined from a random radial angle (0–360 degrees) and a random distance from the stimulus’ center (33–100% stimulus radius). The stimulus changed in shape in visual and multimodal trials. Participants were instructed to hit space bar when the visual stimulus changed in form or when an auditory sound was played in the active blocks. Fifty catch trials were added in this experiment during which there was no visual change and no auditory stimulus to encourage participants to respond only to a visual change or auditory onset.

To calculate the area under the curve, we initially aimed to use the same time window in Experiment 1, but given that the task and the stimulus presentation were different, and the manual and pupil responses were slower, we decided to use the same time window that was used by authors of the study that Experiment 2 was inspired by^23^,1255–1948 ms). This time window captured the peak pupil change in our experiment better than the 500–1200 ms time window that we used in Experiment 1.

## Supplementary information

Supplementary Information

## Data Availability

The data and analyses can be downloaded from the Open Science Framework: https://osf.io/xa6q9/ (Van der Stoep et al., 2020)^54^.

## References

[1] Spence C, Lee J, Van der Stoep N (2017). Responding to sounds from unseen locations: crossmodal attentional orienting in response to sounds presented from the rear. Eur. J. Neurosci..

[2] Van der Stoep N, Nijboer TCW, Van der Stigchel S, Spence C (2015). Multisensory interactions in the depth plane in front and rear space: a review. Neuropsychologia.

[3] *Crossmodal Space and Crossmodal Attention*. (Oxford University Press, Oxford, 2004).

[4] King, A. J. Visual influences on auditory spatial learning. *Philos. Trans. R. Soc. Lond. B Biol. Sci.* **364,** 331–339 (2009). 10.1098/rstb.2008.0230 PMC2674475 18986967

[5] Frens MA, Van Opstal AJ, Van der Willigen RF (1995). Spatial and temporal factors determine auditory-visual interactions in human saccadic eye movements. Percept. Psychophys..

[6] Odegaard B, Wozny DR, Shams L (2015). Biases in visual, auditory, and audiovisual perception of space. PLoS Comput. Biol..

[7] Rohde M, van Dam LCJ, Ernst MO (2016). Statistically optimal multisensory cue integration: a practical tutorial. Multisens. Res..

[8] Alais D, Burr D (2004). The ventriloquist effect results from near-optimal bimodal integration. Curr. Biol..

[9] Ernst MO, Banks MS (2002). Humans integrate visual and haptic information in a statistically optimal fashion. Nature.

[10] Miller J (1982). Divided attention: evidence for coactivation with redundant signals. Cogn Psychol.

[11] Gondan M, Minakata K (2016). A tutorial on testing the race model inequality. Atten. Percept. Psychophys..

[12] Otto TU, Mamassian P (2012). Noise and correlations in parallel perceptual decision making. Curr. Biol..

[13] Meredith MA, Stein BE (1996). Spatial determinants of multisensory integration in cat superior colliculus neurons. J. Neurophysiol..

[14] Wallace MT, Meredith MA, Stein BE (1998). Multisensory integration in the superior colliculus of the alert cat. J. Neurophysiol..

[15] Stein, B. E. & Meredith, M. A. Multisensory integration. Neural and behavioral solutions for dealing with stimuli from different sensory modalities. *Ann. N. Y. Acad. Sci.* **608,** 51–65; discussion 65 (1990). 10.1111/j.1749-6632.1990.tb48891.x 2075959

[16] Bell AH, Meredith MA, Van Opstal AJ, Munoz DP (2005). Crossmodal integration in the primate superior colliculus underlying the preparation and initiation of saccadic eye movements. J. Neurophysiol..

[17] Stein BE, Stanford TR (2008). Multisensory integration: current issues from the perspective of the single neuron. Nat. Rev. Neurosci..

[18] Wang C-A, Boehnke SE, White BJ, Munoz DP (2012). Microstimulation of the monkey superior colliculus induces pupil dilation without evoking saccades. J. Neurosci..

[19] Mathôt S, Van der Stigchel S (2015). New light on the mind’s eye: the pupillary light response as active vision. Curr Dir Psychol Sci.

[20] Naber M, Alvarez GA, Nakayama K (2013). Tracking the allocation of attention using human pupillary oscillations. Front. Psychol..

[21] Naber, M. & Murphy, P. Pupillometric investigation into the speed-accuracy trade-off in a visuo-motor aiming task. *Psychophysiology* e13499 (2019). 10.1111/psyp.13499. 10.1111/psyp.13499 PMC7027463 31736089

[22] Stevenson RA (2014). Identifying and quantifying multisensory integration: a tutorial review. Brain Topogr.

[23] Rigato S, Rieger G, Romei V (2016). Multisensory signalling enhances pupil dilation. Sci. Rep..

[24] Stein BE, Stanford TR, Ramachandran R, Perrault TJ, Rowland BA (2009). Challenges in quantifying multisensory integration: alternative criteria, models, and inverse effectiveness. Exp. Brain Res..

[25] Wang C-A, Boehnke SE, Itti L, Munoz DP (2014). Transient pupil response is modulated by contrast-based saliency. J. Neurosci..

[26] Wang C-A, Blohm G, Huang J, Boehnke SE, Munoz DP (2017). Multisensory integration in orienting behavior: pupil size, microsaccades, and saccades. Biol. Psychol..

[27] Otto TU (2019). RSE-box: An analysis and modelling package to study response times to multiple signals. TQMP.

[28] Miller J (1986). Timecourse of coactivation in bimodal divided attention. Percept. Psychophys..

[29] Miller J (2016). Statistical facilitation and the redundant signals effect: What are race and coactivation models?. Atten. Percept. Psychophys..

[30] Wang C-A, Munoz DP (2015). A circuit for pupil orienting responses: implications for cognitive modulation of pupil size. Curr. Opin. Neurobiol..

[31] Grice GR, Canham L, Gwynne JW (1984). Absence of a redundant-signals effect in a reaction time task with divided attention. Percept. Psychophys..

[32] Calvert, G. A., Spence, C. & Stein, B. E. *The handbook of multisensory process*. (2004).

[33] Stein, B. E. The new handbook of multisensory processing. (2012).

[34] *The neural bases of multisensory processes*. (CRC Press/Taylor & Francis, London, 2012). 22593873

[35] Meredith MA, Stein BE (1986). Visual, auditory, and somatosensory convergence on cells in superior colliculus results in multisensory integration. J. Neurophysiol..

[36] Hughes HC, Reuter-Lorenz PA, Nozawa G, Fendrich R (1994). Visual-auditory interactions in sensorimotor processing: Saccades versus manual responses. J. Exp. Psychol. Hum. Percept. Perform..

[37] Van der Stoep N, Spence C, Nijboer TCW, Van der Stigchel S (2015). On the relative contributions of multisensory integration and crossmodal exogenous spatial attention to multisensory response enhancement. Acta Psychol. (Amst.).

[38] Van der Stoep N, Van der Stigchel S, Nijboer TCW, Van der Smagt MJ (2016). Audiovisual integration in near and far space: effects of changes in distance and stimulus effectiveness. Exp. Brain Res..

[39] Van der Stoep N, Van der Stigchel S, Van Engelen RC, Biesbroek JM, Nijboer TCW (2019). Impairments in Multisensory Integration after Stroke. J. Cogn. Neurosci..

[40] Colonius, H., Wolff, F. H. & Diederich, A. Trimodal race model inequalities in multisensory integration: I. basics. *Front. Psychol.* **8,** 1141 (2017). 10.3389/fpsyg.2017.01141 PMC5504196 28744236

[41] Los SA, Schut MLJ (2008). The effective time course of preparation. Cogn. Psychol..

[42] Los SA, Van der Burg E (2013). Sound speeds vision through preparation, not integration. J. Exp. Psychol. Hum. Percept. Perform..

[43] Gondan M, Lange K, Rösler F, Röder B (2004). The redundant target effect is affected by modality switch costs. Psychon. Bull. Rev..

[44] Otto TU, Dassy B, Mamassian P (2013). Principles of multisensory behavior. J. Neurosci..

[45] Corneil BD, Van Wanrooij M, Munoz DP, Van Opstal AJ (2002). Auditory-visual interactions subserving goal-directed saccades in a complex scene. J. Neurophysiol..

[46] Bailey HD, Mullaney AB, Gibney KD, Kwakye LD (2018). Audiovisual integration varies with target and environment richness in immersive virtual reality. Multisens. Res..

[47] Stein, B. E. & Meredith, A. *The merging of the senses*. (The MIT Press, London, 1993).

[48] Brainard DH (1997). The psychophysics toolbox. Spat Vis.

[49] Kleiner M (2007). What’s new in psychtoolbox-3. Perception.

[50] Leys C, Ley C, Klein O, Bernard P, Licata L (2013). Detecting outliers: do not use standard deviation around the mean, use absolute deviation around the median. J. Exp. Soc. Psychol..

[51] Talsma D, Woldorff MG (2005). Selective attention and multisensory integration: multiple phases of effects on the evoked brain activity. J. Cogn. Neurosci..

[52] Bradley MM, Miccoli L, Escrig MA, Lang PJ (2008). The pupil as a measure of emotional arousal and autonomic activation. Psychophysiology.

[53] Einhäuser W, Koch C, Carter OL (2010). Pupil dilation betrays the timing of decisions. Front. Hum. Neurosci..

[54] Van der Stoep, N., van der Smagt, M., Notaro, C., Spock, Z., & Naber, M. The additive nature of the human multisensory evoked pupil response dataset. 10.17605/OSF.IO/XA6Q9 (2020, December 26). 10.1038/s41598-020-80286-1 PMC7803952 33436889

