# Supplementary material for: The additive nature of the human multisensory evoked pupil response
Source: Sci Rep. 2021 Jan 12;11:707. doi: 10.1038/s41598-020-80286-1 (PMC7803952; doi:10.1038/s41598-020-80286-1)
Supplement: Supplementary file 1 — Supplementary Information [file 41598_2020_80286_MOESM1_ESM.docx]

The additive nature of the human multisensory evoked pupil response

**Supplementary materials**

N. Van der Stoep, N., M. J. Van der Smagt, C. Notaro, Z. Spock, & M. Naber

Department of Experimental Psychology, Helmholtz Institute, Utrecht University, The Netherlands

Corresponding Author:

Nathan Van der Stoep

[N.VanderStoep@uu.nl](mailto:N.VanderStoep@uu.nl)

Langeveld building, Room H0.26

Heidelberglaan 1

3584 CS, Utrecht

The Netherlands

Experiment 1

*Figure S1.* The difference between the pupil responses in the response and the no response block for auditory (red), visual bright (green), and visual dark targets (blue).

*Figure S2.* The average AUC of the pupil trace for all conditions. In contrast with the figures in the main manuscript, here the corrected sum is based on the visual target condition.

*Figure S3.* The average AUC of the pupil trace for all conditions. In contrast with the figures in the main manuscript, here the corrected sum is based on the visual target condition. SumB = sum of auditory and visual bright, cSumAB / cSumAD = sum of auditory and visual bright – the response component based on auditory targets, cSumVB / cSumVD = sum of auditory and visual bright/dark – the response component based on visual targets.


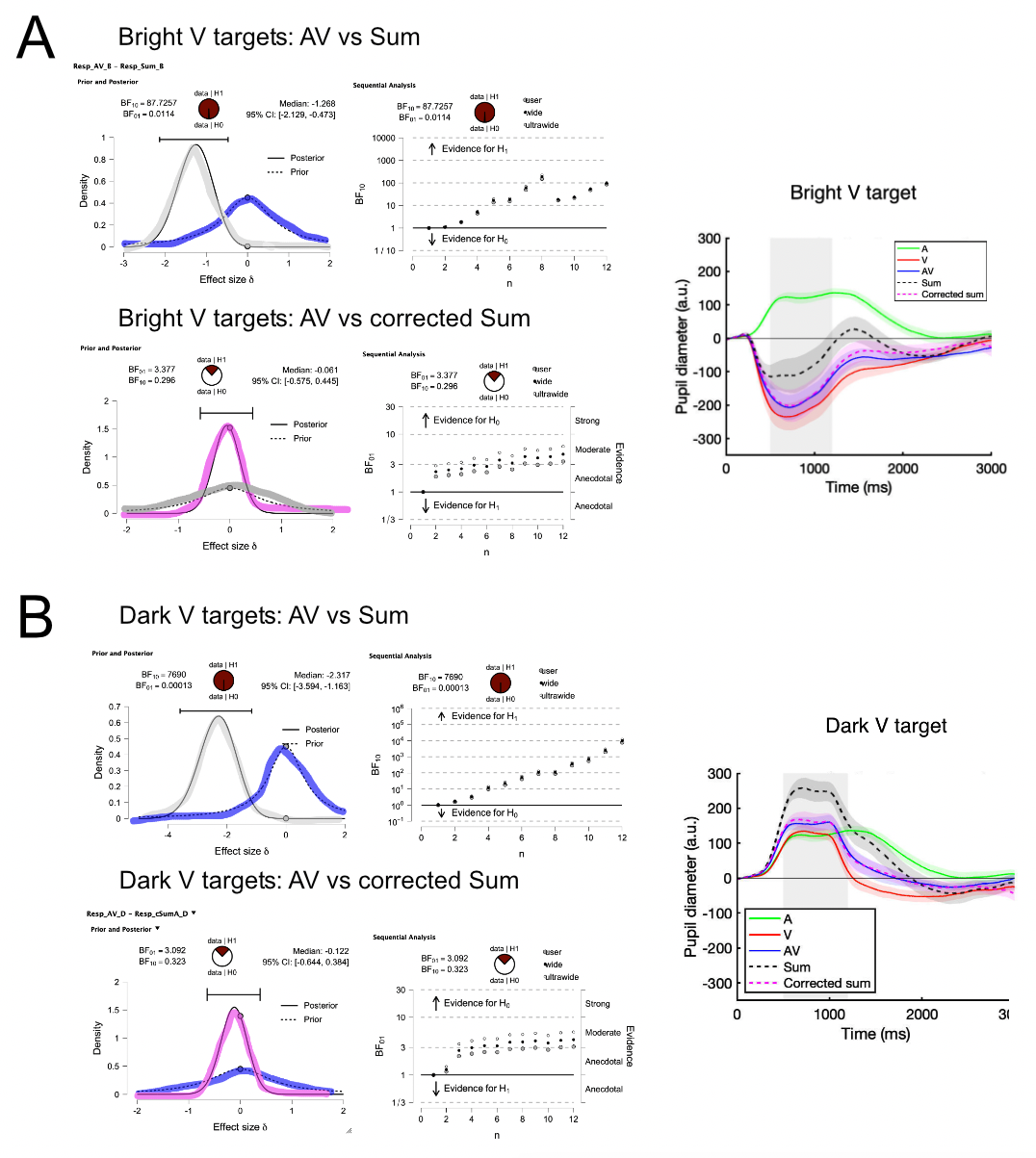


*Figure S4.* A: The results of the Bayesian analysis of the Response block in Experiment 1, comparing the audiovisual (purple/blue), summed (dashed grey), and corrected sum (dashed purple) pupil response AUC for Bright targets including a sequential analysis and pupil response curves for all conditions. B: The results of the Bayesian analysis of the No Response block in Experiment 1, comparing the audiovisual (purple/blue), summed (dashed grey), and corrected sum (dashed purple) pupil response AUC for Dark targets including a sequential analysis and pupil response curves for all conditions.


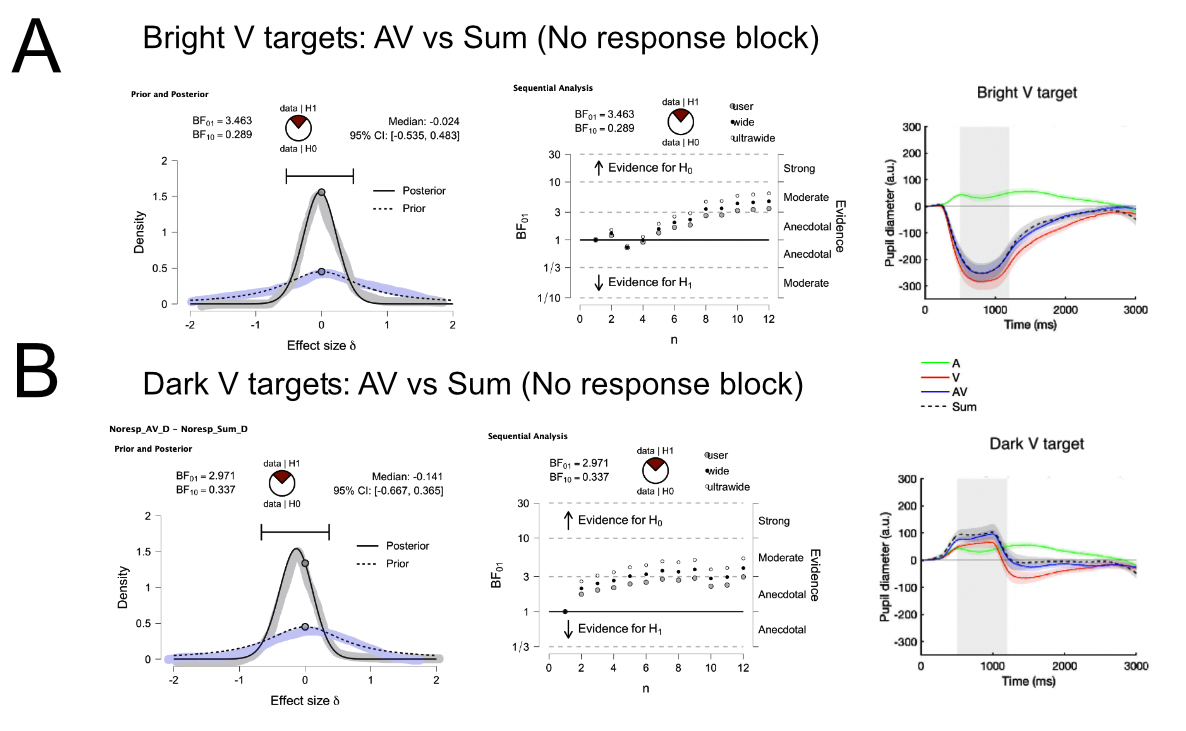


*Figure S5.* A: The results of the Bayesian analysis of the No Response block in Experiment 1, comparing the audiovisual (purple/blue) and summed (dashed grey) pupil response AUC for Bright targets including a sequential analysis and pupil response curves for all conditions. B: The results of the Bayesian analysis of the No Response block in Experiment 1, comparing the audiovisual (purple/blue) and summed (dashed grey) pupil response AUC for Dark targets including a sequential analysis and pupil response curves for all conditions.

Experiment 2

*Figure S6.* The difference between the pupil responses in the response and the no response block for auditory (red), visual (green), and audiovisual targets (blue).

*Figure S7.* The pupil response and average AUC of the pupil trace for all conditions in Experiment 2. In contrast with the figures in the main manuscript, here the corrected sum is based on the visual target condition.

*
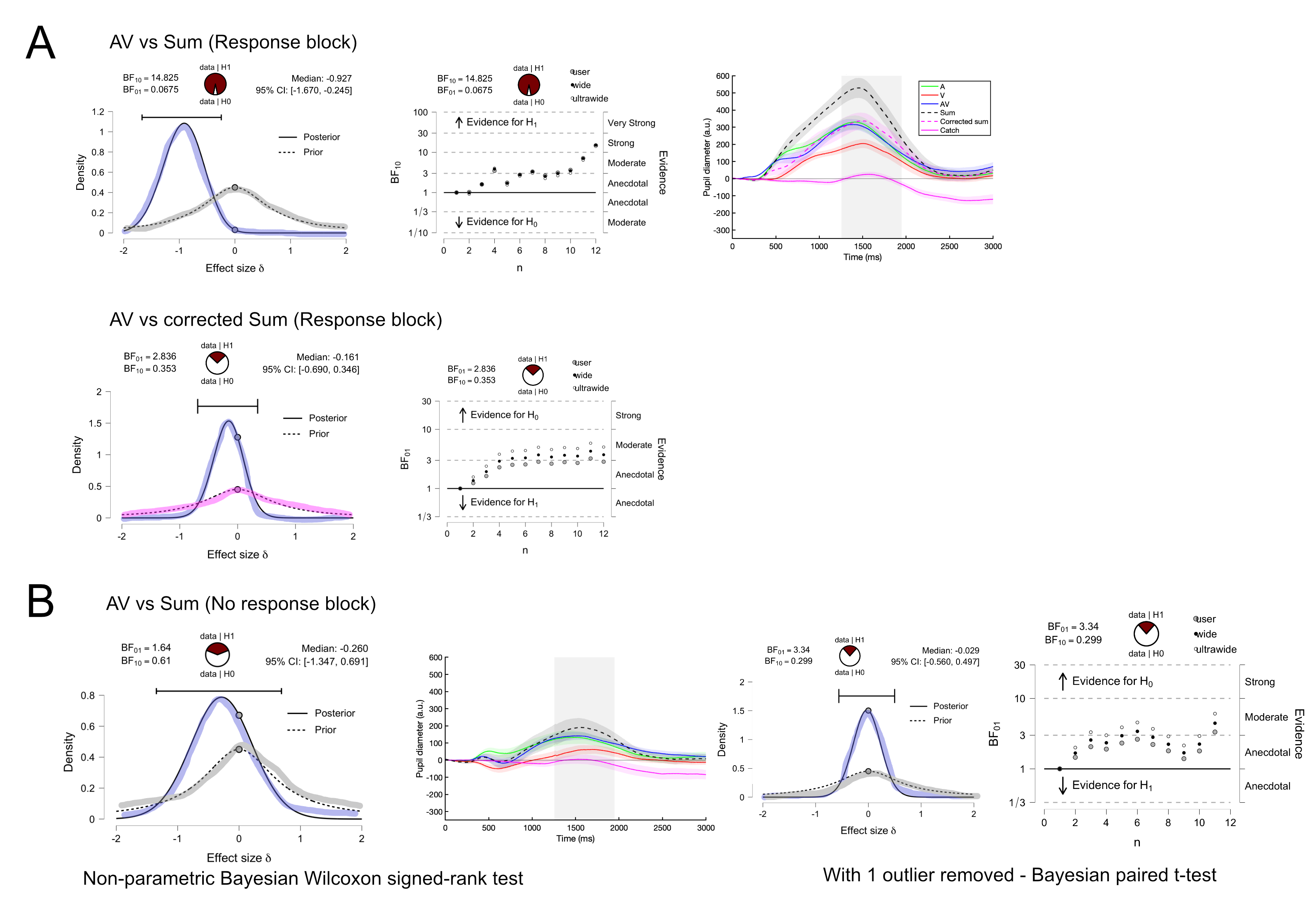
*

*Figure S8.* A: The results of the Bayesian analysis of the Response block in Experiment 2, comparing the audiovisual (purple/blue), summed (dashed grey), and corrected sum (dashed purple) pupil response AUC including a sequential analysis and pupil response curves for all conditions. B: The results of the Bayesian analysis of the No Response block in Experiment 2, comparing the audiovisual (purple/blue) and summed (dashed grey) pupil response AUC including a sequential analysis and pupil response curves for all conditions. Note that the left analysis graph is from a non-parametric Bayesian Wilcoxon signed-rank test due to one participant being an outlier (inter-quartile range cut-off, IQR: i.e. < Q1-1.5 x IQR or > Q3 + 1.5 x IQR). For the purpose of comparison, we also performed a regular Bayesian paired t-test after removing this outlier participant (making the data normally distributed).

*Figure S9.* The nine visual stimuli that were used in Experiment 2.
